# Unusual presentation of cytomegalovirus and varicella-zoster virus infection in a patient with good's syndrome: A case report

**DOI:** 10.1016/j.radcr.2024.09.013

**Published:** 2024-09-26

**Authors:** Payam Tabarsi, Arya Kazemi, Ali Hajihashemi, Mahsa Geravandi

**Affiliations:** a Chronic Respiratory Diseases Research Center, National Research Institute of Tuberculosis and Lung Disease (NRITLD), Shahid Beheshti University of Medical Sciences, Tehran, Iran; b Department of Radiology, Shahid Beheshti University of Medical Sciences, Tehran, Iran; c Department of Radiology, Isfahan University of Medical Sciences, Isfahan, Iran

**Keywords:** Good's syndrome, Cytomegalovirus and Varicella-zoster virus coinfection, Thymoma

## Abstract

The co-infection of cutaneous cytomegalovirus (CMV) and varicella-zoster virus (VZV) is a rare occurrence, particularly in the context of Good's syndrome (GS). This report presents a unique case of a 56-year-old man with GS, characterized by a thymoma, who developed cutaneous CMV and VZV co-infections. We discuss the clinical characteristics, diagnostic process, and treatment of this uncommon manifestation. A 56-year-old man with a mediastinal mass identified as thymoma type A and pleural metastasis, presented with a three-week progressive, painful generalized skin lesions on his face, trunk, and extremities. Upon further examination, multiple extensive vesicles with reddened bases were distributed throughout all regions of his body, some exhibited hemorrhagic characteristics. The histopathologic assessment indicated a herpes virus infection and leukocytoclastic vasculitis. Additionally, Immunohistochemistry staining for CMV highlighted infected endothelial cells. The patient was diagnosed with disseminated cutaneous co-infection of CMV and VZV in the context of Good's syndrome. The patient responded well to a treatment regimen combining intravenous immunoglobulin (IVIG) and ganciclovir therapy, leading to the complete resolution of skin lesions. He was recommended prophylactic treatment and showed significant improvement upon follow-up, ultimately being discharged in a favorable clinical condition. By gaining a better understanding of this condition, healthcare professionals can improve their ability to recognize and treat Good's syndrome effectively. Early identification and effective treatment have a crucial role in enhancing the prognosis.

## Introduction

Good's syndrome, a relatively uncommon immunodeficiency disorder, is primarily characterized by autoimmune manifestations and invasive infections, often observed in older adults. The clinical hallmarks of Good's Syndrome include thymoma, hypogammaglobulinemia, CD4+ T-cell lymphopenia, an inverted CD4+ /CD8+ ratio, and compromised T-cell mitogen proliferative responses [1,2].

There is currently limited knowledge about GS' causes and underlying pathophysiology. It is proposed that an inadequate number of natural killer and CD4 + γδ T cells might contribute to insufficient immune surveillance of tumor growth and ineffective elimination of viral infections [3]. Consequently, patients with GS are highly susceptible to viral infections, including opportunistic pathogens such as herpes simplex, human herpesvirus 8, varicella-zoster virus (VZV), cytomegalovirus (CMV), and Pneumocystis carinii pneumonia (PCP). Among these, VZV is one of the most common herpes viruses causing cutaneous infections in immunosuppressed patients. Reactivations of VZV can be dermatomal but may also disseminate in severely immunodeficient patients. Furthermore, disseminated CMV infection can be life-threatening in immunocompromised patients and can affect multiple organs. CMV infection can cause cutaneous ulcers, often resembling other dermatologic conditions, such as vasculitis. Additionally, GS patients may experience CMV colitis and retinitis as part of their clinical spectrum [4].

In our review of the existing literature, A few reports have addressed the manifestations of CMV infection, particularly CMV pneumonia, retinitis, and uveitis, in the context of GS. Moreover, some studies suggest that patients with GS are more susceptible to VZV infections, such as shingles and Ramsay Hunt syndrome. However, there is a lack of specific case reports on disseminated cutaneous VZV infections and CMV infections in GS patients. Therefore, we present the first case report of Good's Syndrome with disseminated cutaneous co-infection of CMV and VZV, accompanied by a review of the relevant literature.

## Case presentation

**Fig. 1:**
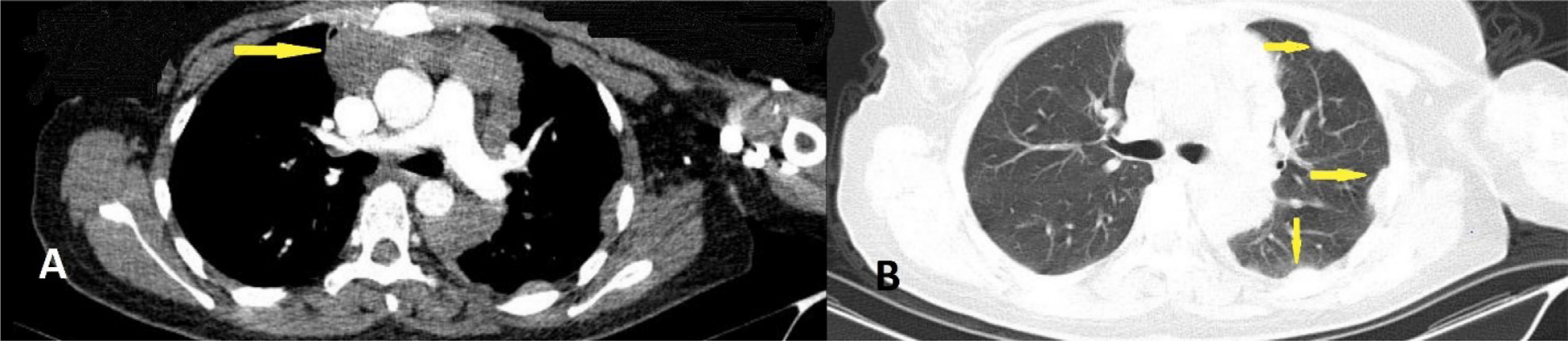
Axial (A, B) chest CT scan with IV contrast. (A): The large lobulated mass in the superior and anterior mediastinal region (yellow arrow). (B): Multiple pleural based lesions in the left hemi thorax, suggestive of pleural metastases (yellow arrows).

A 56-year-old man presented to the emergency department with a three-week history of progressive, painful, generalized skin lesions on his face, trunk, and extremities. These symptoms were followed by the new onset of fever, malaise, fatigue, and night sweats. The patient had no history of tobacco smoking, alcohol consumption, or substance abuse, and he denied recent travel. The patient was diagnosed with a thymoma three years ago, which revealed a 12.5 cm lobulated mass and multiple soft tissue attenuated pleural lesions. The mass was classified as a WHO histologic type A thymoma with pleural metastases (Fig. 1). Following the successful surgical removal of the tumor, the patient underwent chemotherapy and has remained healthy during follow-up, without significant medical problems.

**Fig. 2:**
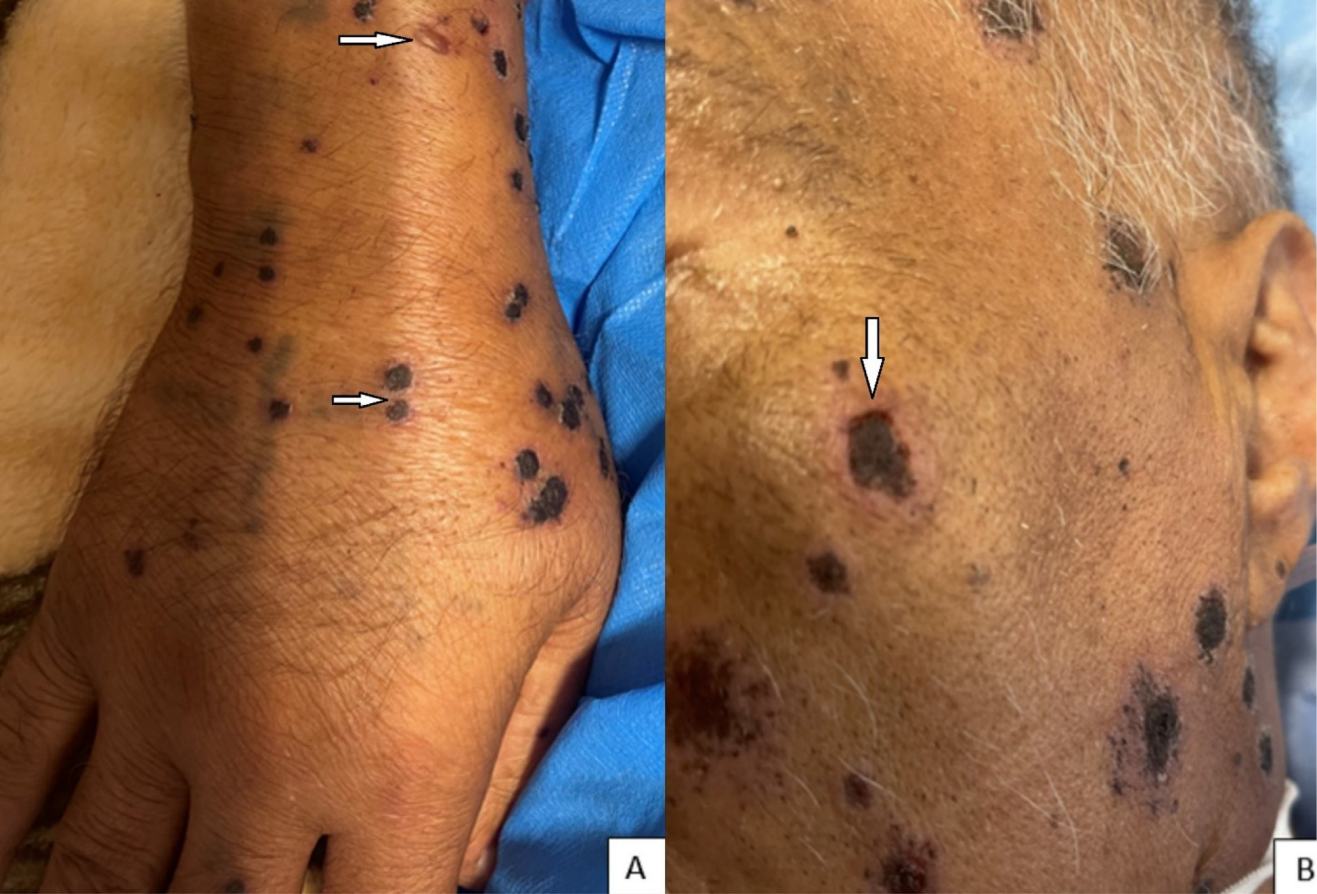
VZV and CMV infections associated with Good's syndrome. **(A):** multiple violet black papules with hemorrhagic crusts on the dorsal side of the hand (VZV isolated)**. (B):** Indurated plaques with hemorrhagic crusts and scalloped border on the face (CMV isolated).

Upon admission, the patient exhibited hemodynamic stability with a blood pressure of 130/100 mmHg and a pulse rate of 80 bpm. He presented with a fever, indicated by an oral temperature of 39.2°C, a respiratory rate of 20 breaths per minute, and an oxygen saturation of 96% on room air. A dermatological evaluation revealed extensive vesicles with reddened bases distributed throughout the body, some of which were hemorrhagic. Additionally, generalized indurated plaques with scalloped borders and violet-black papules were present, accompanied by hemorrhagic crusts (Fig. 2). The remainder of the physical examination, including assessments of the heart, lungs, and neurological system, was unremarkable.

The initial laboratory results indicated an elevated erythrocyte sedimentation rate of 40 mm/h (reference range: 0 to 22 mm/hr) and a C-reactive protein level of 50 mg/L (< 10 mg/L). The complete blood count test findings were hemoglobin of 14.5 g/dL (reference range: 13.8 to 17.2 g/dL), neutrophil-dominant leukocytosis (white blood cells: 10.2 × 10^9^/L, neutrophil count: 85%, Lymphocytes count: 5%, Monocytes count: 2%, Eosinophils count: 1%, Basophils count: 1%) and platelet count of 28.7 × 10**^4^**/µl (13.5 × 10**^4^**/µl to 31.7 × 10**^4^**/µl). The liver and kidney function tests showed normal results. Due to the patient's medical history and the presence of skin lesions, a comprehensive further investigation was conducted. Flow cytometry revealed a decrease in the number of B cells and CD4+ T cells. The average percentage of B cells was less than 1%, while the average percentage of CD4+ T cells was 2.25%. Additionally, CD4/CD8 ratio was reversed. The protein electrophoresis revealed immunoglobulin G (IgG) at 300 mg/dL (reference range: 700-1600 mg/dL) and immunoglobulin A (IgA) at 25 mg/dL (reference range: 70-400 mg/dL). Viral panel tests, including screenings for hepatitis B, hepatitis C, and human immunodeficiency virus (HIV), were negative. Furthermore, a thoracic CT scan revealed no intrathoracic mass lesion, with no indications of thymoma recurrence or pleural metastasis.

**Fig. 3:**
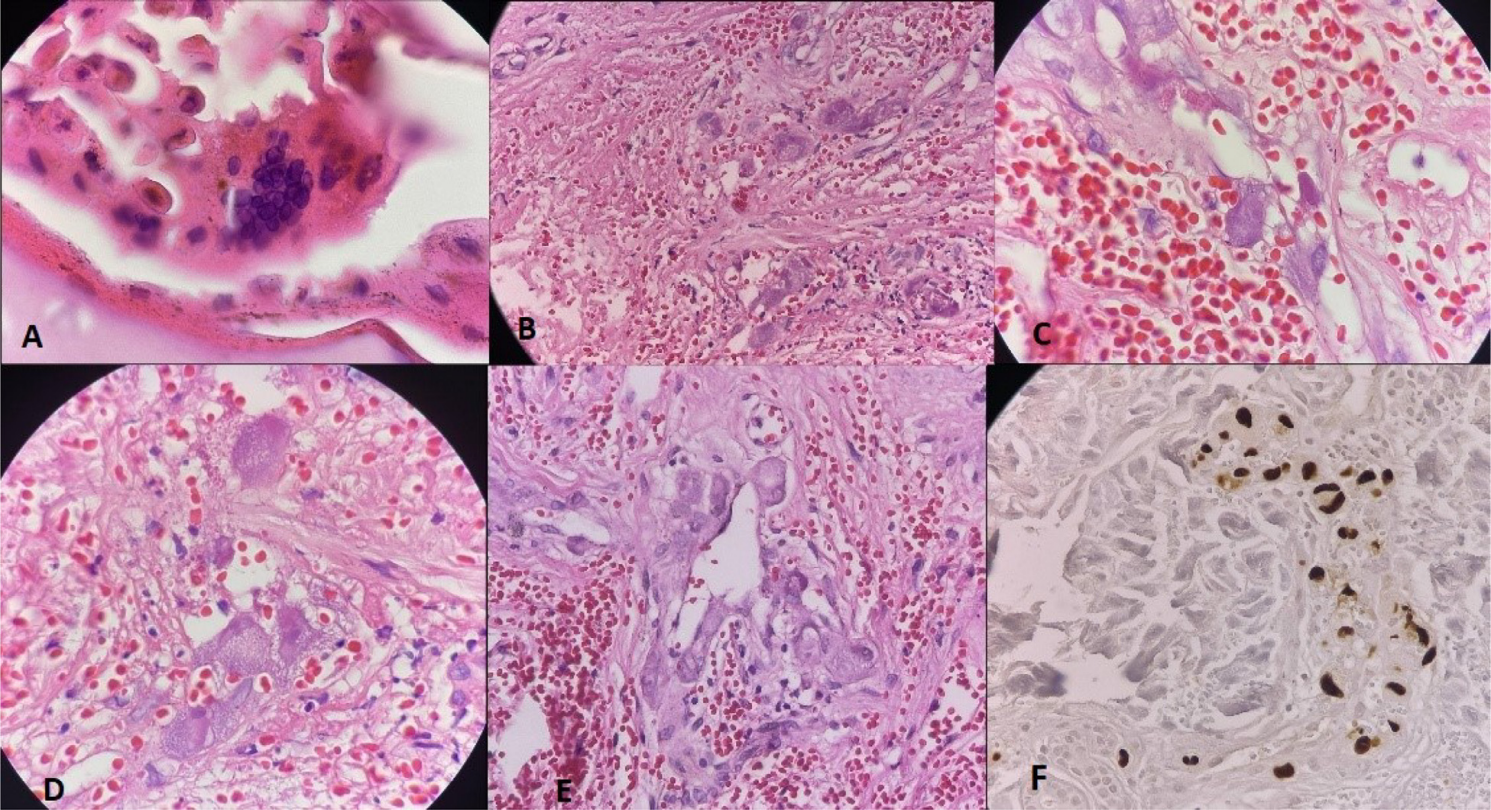
Histopathology of skin lesions: **(A)**: superficial giant keratinocytes with multinucleation, gunmetal gray appearance, and distinct chromatin rim highly suggestive of herpesvirus infection. **(B and C):** Superficial and deep dermis vessels exhibit leukocytoclastic vasculitis. **(D and E)**: Enlarged and atypical endothelial cells with basophilic intranuclear and granular intracytoplasmic inclusions. (Hematoxylin and eosin stain). **(F)**: Immunohistochemistry stain for CMV highlights infected endothelial cells.

Based on the initial findings, a decision was made to perform a skin biopsy. The histopathologic assessment of the skin biopsy showed superficial giant keratinocytes with multinucleation, a gunmetal gray appearance, and a distinct chromatin rim. These characteristics strongly indicated a herpes virus infection. Furthermore, the dermal blood vessels in both the superficial and deep layers exhibited evidence of leukocytoclastic vasculitis. Notably, enlarged and atypical endothelial cells displaying basophilic intranuclear and granular intracytoplasmic inclusions were observed. Immunohistochemistry staining for CMV highlighted infected endothelial cells (Fig. 3). Quantitative real-time PCR analysis of both the fresh biopsy sample and blood samples detected a substantial DNA load of varicella-zoster virus (VZV) and CMV.

Considering these observations (including history of thymoma and immunological abnormalities), a diagnosis of GS was established, further complicated by disseminated cutaneous co-infections CMV and VZV. The patient showed a positive response to a treatment plan that involved intravenous immunoglobulin (IVIG) and ganciclovir. This treatment led to the complete resolution of the skin lesions and a significant improvement in the overall clinical condition of the patient. Following successful management, the patient was recommended to follow a preventive treatment that included valganciclovir, cotrimoxazole, and monthly IVIG infusion therapy. After receiving effective treatment and preventive measures, the patient's condition significantly improved, leading to his discharge from the hospital in a favorable clinical state.

## Discussion

Good's Syndrome is a rare condition affecting individuals in their fourth or fifth decade of life, causing asymptomatic anterior mediastinal masses, including thymoma. GS is characterized by a constellation of immunological abnormalities, such as reduced levels of immunoglobulins IgG, IgM, and IgA, T-cell dysfunction, and an inverted ratio of CD4+ to CD8+. Furthermore, over half of GS patients experience hematological abnormalities including anemia, low white blood cell counts, thrombocytopenia, neutropenia, and eosinophil deficiency. GS is associated with monoclonal gammopathies and T-cell tumors [4,5]. Despite their heightened susceptibility to infections such as bacterial, fungal, viral, and opportunistic infections, concurrent cutaneous infections with both CMV and VZV coinfection remain rare in GS patients. Tarr et al. reported viral infections in 40% of GS patients, with CMV, herpes viruses, and VZV being the most commonly isolated organisms, in descending order of prevalence [6]. Opportunistic infections due to cell-mediated immunity disorders are common in GS, with notable manifestations including CMV-associated colitis, retinitis, and mucocutaneous candida infections. Less prevalent symptoms include skin disorders, urinary tract infections, and gastrointestinal infections [7]. The type and location of infection can impact the prognosis and survival of patients with GS. In the case of concurrent cutaneous CMV and VZV infection, the severity and clinical course depend on various factors, including the individual's immune response and any additional complications. It is noteworthy that only a limited number of publications have reported on CMV and VZV retinitis or pneumonia in the context of GS [[6], [7], [8],[10], [11], [12], [13]].

A case study by Jacox R et al. reported a 54-year-old patient with coexisting thymoma and hypogammaglobulinemia presented with a cough, chest pain, and fever. Radiographic examination revealed diffuse nodular and linear densities indicative of a lung infection. Analysis of the sputum and urine confirmed the presence of CMV, identified by the large inclusion within the fibroblasts' nuclei [8]. Moreover, Mangan KF et al. presented an 80-year-old patient with panhypogammaglobulinemia, an anterior mediastinal tumor, and severe anemia. She had a history of both facial herpes zoster and chronic mucocutaneous candidiasis. The patient was diagnosed with spindle cell thymoma, pure red cell aplasia, and CMV pneumonia following further testing [9]. However, in our case, a man with thymoma and hypogammaglobulinemia presents with cutaneous involvement of CMV and VZV without systematic manifestations.

Ide et al. described a patient with GS who presented with CMV pneumonia. CMV DNA was detected in bronchoalveolar lavage fluid cells via polymerase chain reaction [10]. Furthermore, Yong et al. and Mateo-Montoya et al. described patients aged 50 and 31 years, respectively, with GS presenting with blurred vision due to CMV retinitis [11,12].

In the other study, Inomata et al. reported the case of a 63-year-old patient with GS who had bilateral necrotizing retinitis. This patient tested positive for VZV in a PCR test, indicating atypical VZV retinitis. The patient had a prior history of malignant thymoma that had not metastasized. The treatment approach for this case involved the administration of valacyclovir [13].

Overall, while there have been reports of VZV and CMV infections in patients with GS, the manifestation of these infections may vary among individuals. To the best of our knowledge, this study describes the first case of GS with benign thymoma presenting with cutaneous manifestations of both CMV and VZV. However, further research and clinical studies are needed to provide more specific insights into the occurrence and management of concurrent cutaneous CMV and VZV infections in GS.

## Conclusion

In the current study, we presented a case of cutaneous CMV and VZV co-infection in a patient with GS. Therefore, clinicians should be suspicious of GS when managing patients with a thymoma or mediastinal mass presenting with opportunistic infections. The goal is to enhance awareness surrounding this particular ailment, as early identification and effective treatment can significantly improve prognosis.

## Ethical approval and consent to participate

This case report did not need approval from an ethics committee since it involved one patient. Additionally, informed consent to participate was obtained from the patient.

## Availability of data and materials

All data and materials are available from the corresponding author upon request.

## Authors’ contributions

M.G., A.H., and A.K. planned the study, researched the data, and wrote the manuscript. P.T. reviewed and edited the manuscript.

## Patient consent

Written informed consent for publication was obtained from the patient.
